# A review of excluded groups and non-response in population-based mental health surveys from high-income countries

**DOI:** 10.1007/s00127-023-02488-y

**Published:** 2023-05-22

**Authors:** Eryn Wright, Claudia Pagliaro, Imogen S. Page, Sandra Diminic

**Affiliations:** 1 School of Public Health, The University of Queensland, Herston, Qld 4006 Australia; 2 Queensland Centre for Mental Health Research, Locked Bag 500, Archerfield, Qld 4108 Australia

**Keywords:** Mental health, Population surveys, Epidemiological surveys, Survey methodology

## Abstract

National mental health surveys play a critical role in determining the prevalence of mental disorders in a population and informing service planning. However, current surveys have important limitations, including the exclusion of key vulnerable groups and increasing rates of non-response. This review aims to synthesise information on excluded and undersampled groups in national mental health surveys. We conducted a targeted review of nationally representative adult mental health surveys performed between 2005 and 2019 in high-income OECD countries. Sixteen surveys met our inclusion criteria. The response rate for included surveys ranged between 36.3% and 80.0%. The most frequently excluded groups included people who were homeless, people in hospitals or health facilities and people in correctional facilities. Males and young people were the most commonly underrepresented groups among respondents. Attempts to collect data from non-responders and excluded populations were limited, but suggest that mental health status differs among some of these cohorts. The exclusion of key vulnerable groups and high rates of non-response have important implications for interpreting and using the results of national mental health surveys. Targeted supplementary surveys of excluded or hard-to-reach populations, more inclusive sampling methodologies, and strategies aimed at improving response rates should be considered to strengthen the accuracy and usefulness of survey findings.

## Introduction

Nationally representative mental health surveys play a critical role in providing quality data to help understand and track the mental health of a population. Most contemporary mental health surveys collect data on more than disorder prevalence alone to gain better insight into the impacts of mental disorders and the types of interventions or services required. For example, surveys conducted as part of the World Health Organization (WHO)’s World Mental Health Survey (WMH) Initiative also collect data on disease burden, relevant risk factors, comorbidities, service use and unmet treatment needs.[1].

On a broad scale, global estimates of the burden of mental and other disorders rely on population-based prevalence surveys as a key input. At a national level, these surveys help to shape the public narrative around mental health within a country and play a fundamental role in influencing mental health policy, planning and funding [2]. In fact, planning and costing tools such as the mental health module within the OneHealth systems planning tool (OHT) [3] and Australia’s National Mental Health Service Planning Framework (NMHSPF) [4, 5] draw heavily on estimates generated by population-based prevalence surveys to model mental health care resource requirements.

While such mental health surveys provide critical insight into the mental health status and needs of a population, it is important to recognise their limitations. These include the ongoing exclusion of key vulnerable groups through sampling methodologies and growing rates of non-response. To date, there has been no review synthesising information on the impacts of non-response or excluded populations in large mental health surveys. Understanding these systematically excluded subpopulations is critical to identifying potential underreporting of population mental health needs and to ensure equity of access and tailored service planning for the highest needs groups.

This review aims to (1) identify which groups are commonly excluded from large mental health surveys in high-income OECD (Organisation for Economic Co-operation and Development) countries, (2) identify survey response rates and describe the characteristics of non-responders, and (3) describe any efforts to examine, supplement or adjust for the impacts of non-response and missing or underrepresented populations.

## Methods

We conducted a targeted literature review of national mental health surveys to extract detail on survey sampling methods by drawing on previous systematic reviews of prevalence data.

### Survey search strategy and inclusion criteria

The Global Burden of Disease Study 2019 (GBD 2019) Data Input Sources Tool [6] was used to screen data sources for prevalence of mental disorders in high-income OECD countries published from 2005 onwards. The GBD inputs are obtained through a systematic review of studies and data sources on the prevalence and burden of mental disorders from 204 countries between 1990 and 2019 [7]. Additional information on the GBD review methodology is available online [7].

From this list, sources were included for further consideration if they described a population-based mental health survey that:

- Ended between January 2005 and December 2019, inclusive; included a nationally representative sample of adults; surveyed multiple classes of mental disorders (e.g. depressive disorders, anxiety disorders, substance use disorders, eating disorders, personality disorders, conduct or impulse control disorders, attention-deficit/hyperactivity disorder, autism spectrum disorders, psychotic disorders); and
- was completed as either a stand-alone mental health survey or conducted as part of a general health survey.
- Sources were excluded from further review if the survey they described:
- was limited to only one gender;
- focused on adults in a narrow age cohort only (e.g. young adults, older adults);
- was a follow-up of a previously surveyed cohort and therefore not a random sample from the general population;
- had specific aims other than determining prevalence of mental disorders that otherwise restricted the eligibility criteria;
- focused exclusively on one class of mental disorder, e.g. depressive disorders only;
- restricted the sample to one region within a country;
- sampled populations across multiple countries as part of the same survey.

A list of relevant surveys was then compiled from the included GBD sources. Where multiple iterations of a survey were conducted for different years, only the most recent version included in GBD was selected.

### Search for published survey methods

A Google search was then conducted from May to September 2021 using the name (or the title of the GBD source where no name was given) and year of each survey as search terms to collect academic and grey literature sources associated with each survey, such as methodology reports and results studies. The first 50 search results were scanned for publications that described the survey in their methodology or findings. Where websites or webpages devoted to listing or archiving a survey’s publications were identified in the search results, these were thoroughly checked. Forward and backward snowballing [8] was conducted using references from the original GBD publication(s) and Google search results as the start set. Forward snowballing was performed using the online citation index *scite* [9], while backward snowballing was performed by hand until saturation was reached (i.e. no new relevant sources were identified). Google searching and snowballing was preferred to a search of scientific databases given that a large proportion of the data of interest lay in grey literature. Government and non-government reports, government webpages, journal articles and manuscripts were reviewed. Online translations of non-English sources were considered for review if they could be verified by a fluent language reader. News articles, commentaries, blog posts, abstracts only and non-government webpages were excluded.

A full-text review of all relevant publications was conducted to identify the primary source of data for the survey methodology. Where multiple publications described the methodology in comparable detail, the earliest published or available resource was selected. Supplementary sources were also included where they provided additional information on: (1) the survey design; (2) survey response rate; (3) excluded populations; (4) characteristics associated with response as judged by the authors of relevant sources; (5) survey adjustments for excluded or underrepresented populations; (6) supplementary or parallel mental health surveys of non-responders or excluded populations; or (7) additional analyses on the association between survey response and disorder prevalence. Where supplementary surveys or additional analyses were explicitly identified in the primary publication, an additional search was conducted using the same Google search and snowballing method to identify relevant publications.

Supplementary non-response surveys were included for data extraction if they collected data on mental health, including symptom measures or diagnosis history. Since resource limitations may restrict the number of additional surveys conducted over time, supplementary surveys of excluded populations conducted for both past and current iterations of the primary survey (up to a maximum of two) were included for data extraction so long as they collected information on mental health, including a diagnostic interview, symptom measures or diagnosis history. Non-response and supplementary surveys focused solely on substance use disorders, mental health treatment or self-harm, suicide attempts and suicidal ideation were not included.

### Data extraction and synthesis

Survey information extracted from relevant sources included country, survey year, survey design, sample size, psychiatric disorders of interest, psychological assessment instruments and response rate. Information extracted on psychiatric disorders excluded indicators of self-harm; suicidal thoughts; suicide attempts; smoking; use of tobacco products; nicotine use, dependence or withdrawal; alcohol or drug use where this was not characterised as harmful, problematic or a substance use disorder; and other general health and well-being measures (e.g. quality of life). Next, data were extracted to describe populations that were identified as underrepresented or less likely to respond, as well as groups that were excluded from participating in the survey because of the sampling methodology or eligibility criteria.

Finally, information was extracted from the relevant literature on additional efforts undertaken to survey non-responders or excluded groups, or to investigate the impacts of response rate on prevalence estimates. This included data on the general design and methodology of the supplementary survey or analysis as well as the year, sample size, psychological assessment measures used, response rate and key findings.

Where relevant data were missing from existing literature or questions remained regarding the sampling methodology, exclusion criteria or supplementary surveys, up to three contributing authors of included sources were contacted for more information.

Following a preliminary review of the data, categories for frequently excluded groups were established through an iterative process, beginning with the categories identified in the source literature and grouping these based on similar characteristics and reasons for exclusion. This process facilitated comparisons between surveys. The data extraction and synthesis were conducted and reviewed by EW, IP and CP.

## Results

### Survey characteristics

**Fig. 1:**
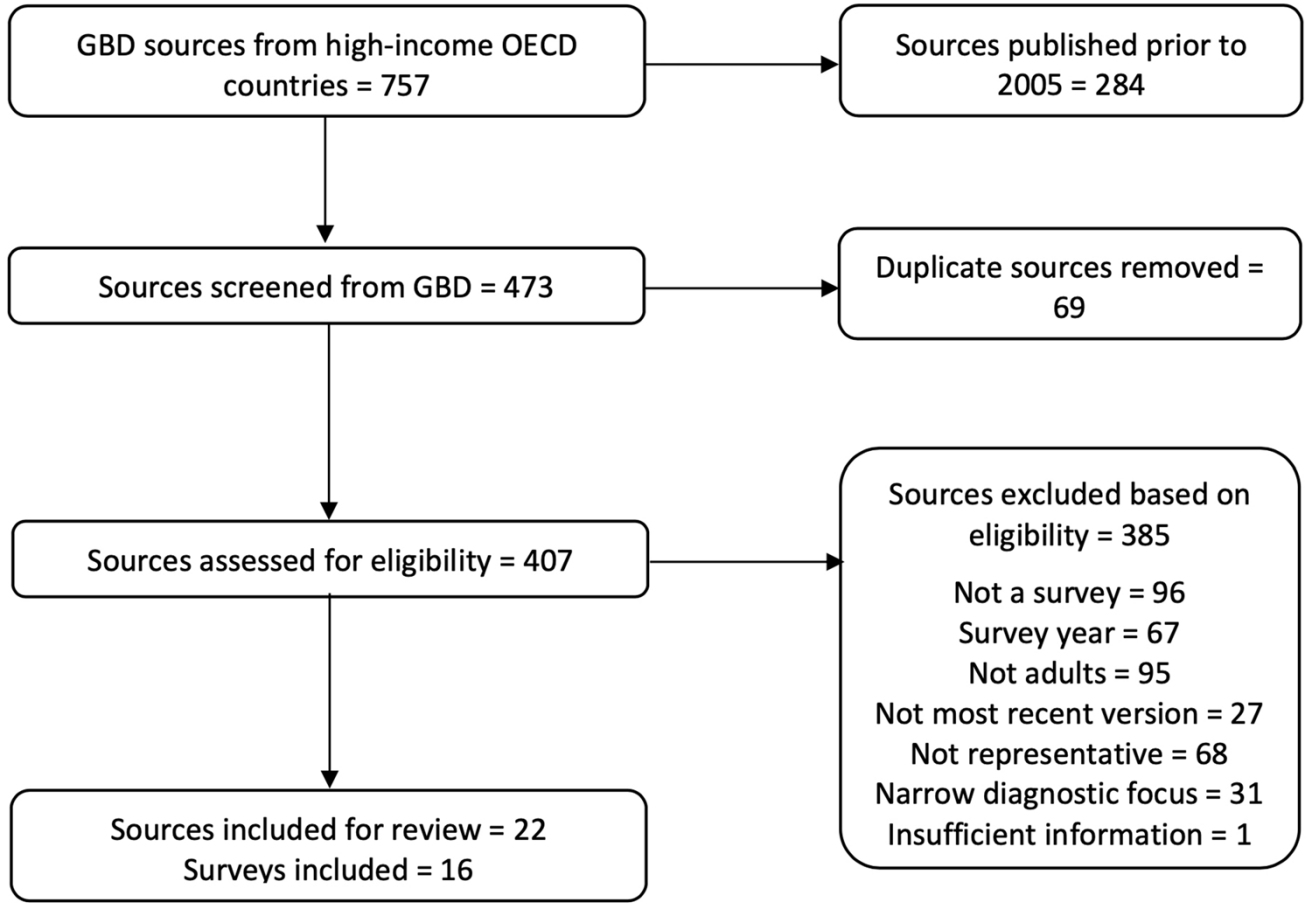
Identification of national mental health surveys [10]

Twenty-two sources from the GBD Data Input Sources Tool described surveys that met our criteria. After accounting for sources that used the same survey, 16 individual surveys [11–29] conducted in 14 countries were included for analysis (Figure 1). Table 1 provides an overview of the methods for each included survey. Fourteen surveys sampled adults only [11–26], while 2 surveys also included children and adolescents under the age of 15 years [27–29]. Fifteen of the surveys were conducted in person [11–29] and 2 was mailed to participants [21], who could mail back the completed survey or complete it online. Three surveys explicitly described attempts to account for the possible underrepresentation of specific groups in their sampling methodologies [11, 13, 20, 27, 29], while 14 applied post-stratification weighting to adjust for sampling bias and non-response and improve representativeness [11–17, 19–29]. All surveys included measures of anxiety and depressive disorders [11–29], 4 included psychotic disorders [12, 14, 19, 20, 26], 2 included personality disorders [12, 14, 19], 2 included conduct or impulse control disorders [19, 22], 1 included autism spectrum disorders [12, 14] and 13 included substance use disorders [11–16, 18–20, 22–29]. All 13 surveys that included substance use disorders included measures of alcohol use disorders [11–16, 18–20, 22, 24–29], while 10 also collected information on other drug use disorders [11–15, 18, 19, 20, 22, 24, 27, 28, 29]. Most surveys were conducted by government agencies and/or academic collaboratives.

**Table 1:** Overview of included nationally representative adult mental health surveys

Country	Survey	Year	Design	Sample	Disorders	Instruments	Response rate	Weighting and adjustments	Characteristics associated with response
Australia	2007 National Survey of Mental Health and Wellbeing (NSMHWB) [11, 13]	2007	Cross-sectional face-to-face survey of usual residents of private dwellings in Australia aged 16–85 years. Younger people (16–24 years) and older people (65–85 years) were given a higher chance of selection within households to increase the representation of these groups	8,841	Anxiety disorders (panic disorder, agoraphobia, social phobia, GAD, OCD, PTSD), affective disorders (depressive episode, dysthymia, BP), and SUDs (dependence on and harmful use of alcohol and drug use disorders including cannabis, sedatives, stimulants and opioids)	CIDI V3.0 K10	60%	Data were weighted to account for the probability of selection, population estimates and household demographics	Older people aged 65–85 years and younger people aged 16–24 years were overrepresented in the survey compared to the national population. Older people were also more likely to respond if selected
Canada	Canadian Community Health Survey- Mental Health (CCHS-MH) [15]	2012	Cross-sectional household survey of individuals aged ≥ 15 years living in Canadian provinces. Participation was face to face or by telephone	25,113	GAD, MDE, BP, and SUDs (abuse of and dependence on alcohol, cannabis or other drugs)	CIDI V3.0 K6	68.9%	Sampling weights were applied to ensure that analyses were representative of the general population	Not reported
England	Adult Psychiatric Morbidity Survey (APMS) 2014 [12, 14]	2014	Cross-sectional survey of individuals aged ≥ 16 years living in private households in England	7,546	Anxiety disorders (GAD, OCD, panic disorders, PTSD and phobias), depressive episode, other common mental disorders not specified, ADHD, SUDs (AUDs and dependence on cannabis, amphetamines, cocaine, crack, ecstasy, heroin, methadone, tranquillisers or volatile substances), psychotic disorder, BPD, ASPD, any personality disorder, ASD and BP	AQ-20 ADOS ASRS AUDIT CIS-R DIS (5 questions on drug dependence) MDQ PCL-C PSQ SADQ SAPAS SCAN V2.1 SCID-II	57%	Data were weighted to account for selection bias and non-response. Disorder-specific weighting was applied for psychotic disorder and ASD in phase 2 to account for disorder-specific probabilities of selection	Males and young people aged 16–24 years were underrepresented when compared to the national population
Greece	The 2009–2010 Psychiatric Morbidity Survey [16]	2009–2010	Cross-sectional face-to-face survey of residents of Greece (excluding residents of Crete) aged 18–70 years living in private households	4,894	Anxiety disorders (GAD, phobias, panic disorder and OCD), depressive episode, mixed anxiety and depression disorder and harmful alcohol use	AUDIT CIS-R	54%	Data were weighted to account for the sampling design and non-response	Sex and age distribution of the sample were representative of the national population. However, women and people aged 40–55 years were less likely to respond if selected
Ireland	Survey of Lifestyle Attitudes and Nutrition (SLAN) 2007 [17]	2007	Cross-sectional face-to-face survey of Irish citizens and non-Irish national residents aged ≥ 18 years living in private households in the Republic of Ireland	10,364	Probable MDD and GAD	CIDI-SF V1.1 MHI-5	62%	Survey weights were applied to ensure that estimates were representative of the general population	Men and younger single adults were underrepresented among respondents when compared to the national population
Japan	World Mental Health Japan 2^nd^ Survey (WMHJ2) [18]	2013–2015	Cross-sectional face-to-face household survey of Japanese residents aged 20 to 75 years	2,450	Anxiety disorders (agoraphobia, GAD, panic disorder, social phobia, PTSD), mood disorders (MDD, BP I and II, dysthymia); and SUDs (alcohol abuse with/without dependence and drug abuse with/without dependence)	CIDI V3.0	43.4%	Not reported	Compared to the national population, respondents were more likely to be married and to have higher education; however, age and gender distribution were representative
The Netherlands	The Netherlands Mental Health Survey and Incidence Study (NEMESIS-2) [19]	2007–2009	Cross-sectional face-to-face survey of people aged 18–64 years residing in private households in the country’s four largest cities and 184 randomly sampled municipalities (of 443)	6,646	Anxiety disorders (panic disorder, agoraphobia, social phobia, specific phobia, GAD), mood disorders (major depression, dysthymia, BP), SUDs (alcohol abuse and dependence, and drug abuse and dependence), impulse control disorders (ADHD, conduct disorder, oppositional-defiant disorder), schizophrenia and ASPD	CIDI V3.0 CIDI 1.1 (adapted schizophrenia section) IPDE	65.1%	Post-stratification weights were applied to account for different response rates between population groups and selection probability	Compared to the national population, males, younger people (especially aged 18–24 years of age), people with higher secondary education, those who were not in paid employment, people of Turkish and Moroccan origin and people living in bigger towns were underrepresented, while people living alone and those with lower secondary education were overrepresented Non-responders were more likely to be male and less likely to be of non-Dutch origin, aged 18–24 years or living alone
New Zealand	The New Zealand Health Survey (NZHS) [27, 29]	2016–2017	Cross-sectional face-to-face survey of usual New Zealand residents, including residents of aged care facilities and students with fixed home addresses residing in university hostels and boarding schools. One adult (≥ 15 years) and one child (0–14 years) were selected from each household. Electoral roll and census data were used to increase the sample sizes of Māori, Pacific and Asian groups	13,598 adults	Anxiety disorder, depressive disorder and risk of problematic substance use (alcohol, amphetamines, cannabis, cocaine, hallucinogens, inhalants, opioids, sedatives and other drugs)	ASSIST K10 PHQ-SADS	80%	A calibrated weighting method was used to account for selection bias and to correct for any discrepancies between the sample and the general population	Not reported
Northern Ireland	Northern Ireland Study of Health and Stress (NISHS) [22]	2004–2008	Cross-sectional face-to-face household survey of people ≥ 18 years in Northern Ireland	4,340	Anxiety disorders (panic disorder, GAD, social phobia, specific phobia, agoraphobia without panic, PTSD, OCD, adult separation anxiety disorder and any anxiety disorder), mood disorders (MDD, dysthymia, BP and any mood disorder), impulse control disorders (oppositional-defiant disorder, conduct disorder, ADHD and IED) and SUDs (alcohol abuse and dependence, and drug abuse and dependence)	CIDI V3.0	68.4%	Data were weighted to account for the probability of selection within households, non-response and socio-demographic variations from the general population. Standard errors were then used to adjust for data weighting and clustering	Not reported
Poland	Epidemiology of mental disorders and access to mental health care—EZOP [24]	2010–2011	Cross-sectional face-to-face survey of Polish citizens aged 18–64 years	10,081	Anxiety disorders (GAD, panic attacks, panic disorder, specific phobia and social phobia), mood disorders (MDD, minor depressive disorder, dysthymia, BP I and II, mania and hypomania) and SUDs (alcohol abuse and dependence, and psychoactive substance abuse and dependence)	CIDI V3.0	50.4%	Post-stratification weights were applied to account for variations in sex, age, place of residence and province compared to the general population	Response rates were highest in rural areas and decreased as population size increased in sampled areas
Portugal	The Portuguese National Mental Health Study (NMHS) [25]	2008–2009	Cross-sectional face-to-face household survey of people aged ≥ 18 years residing in permanent private dwellings in mainland Portugal	3,849	Anxiety disorders (panic disorder, GAD, social phobia, specific phobia, agoraphobia without panic disorder, PTSD, OCD and adult separation anxiety), mood disorders (MDD, dysthymia and BP I and II), and AUDs (alcohol abuse with/without dependence)	CIDI V3.0 WHODAS-II	57.3%	Data were weighted to account for selection probability, non-response,and differences in socio-demographic and geographic distribution compared to the general population	Females and people aged 35–64 years of age were overrepresented in the sample compared to the national population
South Korea	The Korean Epidemiologic Catchment Area Study 2011 (KECA-2011) [26]	2011	Cross-sectional face-to-face household survey of Korean adults aged 18–74 years	6,022	Anxiety disorders (OCD, PTSD, panic disorder, agoraphobia, social phobia, GAD, specific phobia), mood disorders (MDD, dysthymia, BP), psychotic disorders (schizophrenia, schizophreniform disorder, schizoaffective disorder, delusional disorder, brief psychotic disorder) and AUDs (alcohol abuse and dependence)	K-CIDI V2.1	78.7%	Data were weighted to approximate national age and sex distributions	Not reported
	Korean Headache–Sleep Study (KHSS) [23]	2011–2012	Cross-sectional face-to-face survey of the adult Korean population aged 19–69 years	2,695	Anxiety and depression	Goldberg Anxiety Scale PHQ-9	36.3%	Data were weighted to adjust for differences in gender, age group, size of residential area and educational level compared to the general population	Not reported
Sweden	Depression, anxiety and their comorbidity in the Swedish […] [21]	2009	Cross-sectional survey mailed to Swedish residents including citizens and non-citizens aged 18–70 years	3,001	Anxiety, depression, MDD and GAD	GAD-7 GAD-Q-IV PHQ-9	44.3%	Not reported	The rate of post-secondary education was higher among survey respondents than the national population
The United States	2014 National Survey of Drug Use and Health (NSDUH) [28]	2014	Cross-sectional survey of civilian non-institutionalised populations aged ≥ 12 years residing in private households and non-institutional group quarters (e.g. shelters, boarding houses, college dormitories, migratory workers’ camps and halfway houses)	67,901 incl. 50,855 adults	MDE and SUDs including AUDs and drug use disorders (cannabis, cocaine, heroin, hallucinogens, inhalants and the nonmedical use of prescription pain reliever drugs)	K6 WHODAS	58.3%	Analysis weights were created so that estimates were representative of the target population	Not reported
	National Epidemiologic Survey on Alcohol and Related Conditions-III (NESARC-III) [20]	2012–2013	Cross-sectional survey of the non-institutionalised civilian adult population aged ≥ 18 years living in private households and certain group quarters including group homes and workers’ dormitories in the USA. African American, Hispanic and Asian populations were oversampled and eligible individuals were offered a monetary incentive to participate	36,309	Anxiety disorders (panic disorder, social phobia, specific phobia, GAD), mood disorders (MDD, BP type I or II), PTSD, psychotic disorder (schizophrenia or a psychotic illness or episode) and SUDs including AUD and drug use disorders (cannabis, club drugs, cocaine, amphetamine, hallucinogen, heroin, opioid, sedative, tranquilliser, solvent or inhalant)	AUDADIS-5	60.1%	Data were adjusted for oversampling and non-response and weighted to be representative of the general US population	Survey coverage was higher for females, and Black and Hispanic people and lower for males and White people

*ADHD* attention-deficit hyperactivity disorder, *ADOS* Autism Diagnostic Observation Schedule, *AQ-20* 20-Item Autism Quotient, *ASD* autism spectrum disorder, *ASRS* Adult ADHD Self-Report Scale, *ASSIST* Alcohol, Smoking and Substance Involvement Screening Test, *AUD* alcohol use disorder, *AUDADIS-5* the Alcohol Use Disorder and Associated Disabilities Interview Schedule-5, *AUDIT* Alcohol Use Disorders Identification Test, *BP* bipolar disorder, *CIDI 1.1* Composite International Diagnostic Interview, version 1.1, *CIDI V3.0* Composite International Diagnostic Interview, version 3.0, CIDI-*SF* Composite International Diagnostic Interview-Short Form, *CIS-R* Clinical Interview Schedule-Revised, *DIS* Diagnostic Interview Schedule, *GAD* generalised anxiety disorder, *GAD-7* Generalized Anxiety Disorder Scale-7, *GAD-Q-IV* Generalized Anxiety Disorder Questionnaire-IV, *K6* the Kessler Psychological Distress Scale-6, *K10* Kessler Psychological Distress Scale-10, *K-CIDI V2.1* Korean Composite International Diagnostic Interview, version 2.1, *IED* intermittent explosive disorder, *IPDE* International Personality Disorder Examination, *MDD* major depressive disorder, *MDE* major depressive episode, *MDQ* Mood Disorder Questionnaire, *MHI-5* Mental Health Inventory-5, *OCD* obsessive compulsive disorder, *PCL-C* PTSD checklist-civilian, *PHQ-9* Patient Health Questionnaire-9, PHQ-SADS: Patient Health Questionnaire-Somatic, Anxiety, and Depressive Symptoms, *PSQ* Psychosis Screening Questionnaire, *PTSD* posttraumatic stress disorder, *SADQ* Severity Of Alcohol Dependence Questionnaire, *SAPAS* Standardised Assessment of Personality: Abbreviated Scale, *SCAN V2.1* Schedule for Clinical Assessment In Neuropsychiatry, version 2.1, *SCID-II *Structured Clinical Interview for DSM-IV Axis II Disorders, *SUDs* substance use disorders, *US* United States, *WHODAS* World Health Organization Disability Assessment Schedule

### Excluded populations

Details about inclusion and exclusion criteria were identified for all included surveys; however, the amount of information provided was highly variable. Based on available published data, we identified 13 populations or groups that were commonly excluded from national mental health surveys (Table 2). These populations are listed below in order from most to least commonly excluded:

- Homeless people with no fixed address (14 of 16 surveys).
- People in hospitals and health facilities (14 of 16 surveys.)
- People residing in correctional facilities (14 of 16 surveys).
- People in residential care facilities (12 of 16 surveys).
- Military personnel on base or abroad (12 of 16 surveys).
- Non-local language speakers (11 of 16 surveys).
- People residing in temporary housing (11 of 16 surveys).
- Short-term overseas visitors (11 of 16 surveys).
- People living on islands, in remote areas or in specific territories (9 of 16 surveys).
- People living in other non-private dwellings (e.g. migratory worker dormitories, detention facilities, monasteries, etc.) (8 of 16 surveys).
- People living in educational institutions (7 of 16 surveys).
- Non-residents (7 of 16 surveys).
- People with a cognitive impairment (7 of 16 surveys).

**Table 2:** Populations covered in national mental health surveys

Country	Survey	Year	Populations												
			Non-local language speakers	Non-residents	Short-term overseas visitors	Homeless people with no fixed address	People living in temporary housing	Residential care facilities	Hospitals and health facilities	Correctional facilities	Military personnel on base or abroad	Islands, remote areas or specific territories	People with a cognitive impairment	People living in educational institutions	Other non-private dwellings
Australia	NSMHWB [11, 13, 36]	2007	X	✓	X	X	X	X	X	X	–	X	X	–	X
Canada	CCHS-MH [15, 34]	2012	X	✓	✓	X	** ± **	X	X	X	X	X	Ø	** ± **	X
England	APMS 2014 [14, 30, 37]	2014	X	✓	X	X	X	X	X	X	X	**•**	** ± **	X	X
Greece	The 2009–2010 Psychiatric Morbidity Survey [16]	2009–10	–	–	X	X	X	X	X	X	X	X	–	X	X
Ireland	SLAN 2007 [17]	2007	–	X	X	X	X	X	X	X	X	–	–	X	X
Japan	WMHJ2 [18, 38, 39]	2013–15	X	X	X	X	X	X	X	X	X	X	X	✓	X
The Netherlands	NEMESIS-2 [19]	2007–9	X	–	X	X	X	X	X	X	X	–	–	X	–
New Zealand	NZHS [27]	2016–17	X	X	X	X	X		X	X	X	X	X	✓	–
Northern Ireland	NISHS [22, 32]	2004–8	X	X	X	X	X	X	X	X	X	–	X	X	-
Poland	EZOP [24, 31, 33]	2010–11	X	X	X	X	X	X	X	X	X	✓	X	X	X
Portugal	NMHS [25, 40]	2008–9	X	X	X	X	X	X	X	X	X	X	X	X	X
South Korea	KECA-2011 [26]	2011	X	X	X	X	–	X	X	X					
	KHSS [23]	2011–12	–	–	–	–	–	–	–	–	–	X	–	–	–
Sweden	Depression, anxiety and their comorbidity […] [21]	2009	X	–	–	–	–	–	–	–	–	–	–	–	–
The United States	2014 NSDUH [28, 35]	2014	✓	✓	✓	X	✓	X	X	X	X	X	Ø	✓	✓
	NESARC-III [20, 41]	2012–13	✓	–	–	X	X	–	X	X	X	X	X	✓	✓
		Total excluded	**11**	**7**	**11**	**14**	**11**	**12**	**14**	**14**	**12**	**9**	**7**	**7**	**8**
		Total included	**2**	**4**	**2**	**0**	**1**	**1**	**0**	**0**	**0**	**1**	**0**	**4**	**2**

X excluded, ✓ included, ± case-by-case basis reliant on other factors, **•** not applicable, – no information identified in the literature, Ø  no exclusion or inclusion criteria included in the survey design.

*APMS* Adult Psychiatric Morbidity Survey, *CCHS-MH* Canadian Community Health Survey-Mental Health, *EZOP* epidemiology of mental disorders and access to mental health care, *KECA-2011* the Korean Epidemiologic Catchment Area Study 2011, *KHSS* Korean Headache–Sleep Study, *NEMESIS-2* the Netherlands Mental Health Survey and Incidence Study, *NESARC-III* National Epidemiologic Survey on Alcohol and Related Conditions-III, *NISHS* Northern Ireland Study of Health and Stress, *NMHS* the Portuguese National Mental Health Study, *NSDUH* National Survey of Drug Use and Health, *NSMHWB* National Survey of Mental Health and Wellbeing, *NZHS* the New Zealand Health Survey, *SLAN* Survey of Lifestyle Attitudes and Nutrition, *WMHJ2* World Mental Health Japan 2^nd^ Survey

Information was missing and could not be obtained on the inclusion or exclusion of some of these groups for ten of the surveys, two of which only had data available on the eligibility of one population (see Table 2).

It is worth noting that many surveys only sampled people living in private dwellings, and this often constituted the reason some groups were or may have been excluded. Furthermore, the location-based criteria were often only applicable to the time period in which the fieldwork was conducted and/or whether the individual had a fixed residential address [19, 30, 31]. For instance, a student with a fixed address outside of their educational institution would have been eligible for the 2014 survey in England [30] or the 2010–2011 survey in Poland [31] if they had returned home during the sampling period in that area.

Fewer than half of the surveys had literature that discussed the eligibility of people with a cognitive impairment [13, 20, 25, 29, 32, 33]. In two instances where authors were able to be contacted, it was suggested that while there was no formal cognitive assessment conducted as part of the sampling process, there would likely be a degree of self-selection or implicit exclusion where individuals were unable to understand and respond to the survey questions [34, 35]. In the case of the Adult Psychiatric Morbidity Survey (APMS) conducted in England in 2014, personal communication with the survey team stated that individuals with a cognitive impairment were likely to be excluded unless the impairment was mild [30]. As such, we considered that this exclusion criterion was applied on a case-by-case basis.

### Response rate, underrepresented groups and non-responder characteristics

Survey response rates ranged from 36.3% [23] to 80% [27] (Table 1). Ten of the included surveys had sources that compared the characteristics of people in the survey sample to those of the general population [11, 13, 14, 16–21, 25, 26]. Analysis of these findings showed that males [14, 17, 19, 20, 25] and younger adult cohorts [14, 17, 19] were the most commonly underrepresented groups. Literature on four surveys also reported on factors associated with response rate [13, 16, 19, 24]. However, no common factors influencing response rate were identified between the surveys.

### Supplementary surveys of excluded populations

Attempts to capture excluded populations through supplementary surveys were identified for four of the primary surveys included in our analysis (Table 3):

- The 2007 NSMHWB in Australia
  - Supplementary survey population: people living in rural and remote areas [42–46].
- The Canadian Community Health Survey- Mental Health (CCHS-MH).
  - Supplementary survey population: military personnel [47–50].
- The APMS 2014 in England.
  - Supplementary survey populations: ethnic minorities [51], homeless adults [52], people in correctional facilities [53] and residents of institutions catering to people with mental illness [54].
- The 2014 NSDUH in the USA.
  - Supplementary survey populations: military personnel [55–57] and people in correctional facilities [58–61].

**Table 3:** Supplementary surveys of populations excluded from national mental health surveys

Country	Primary survey (year)	Supplementary survey target population	Description of supplementary survey or analysis	Instrument(s)	Year	Sample	Response rate	Findings
Australia	NSMHWB (2007)	Rural and remote populations [42–46]	The Australian Rural Mental Health Study (ARMHS) was a 5-year longitudinal population study of randomly selected people aged ≥ 18 years living in private households in non-metropolitan areas of New South Wales, 28% of whom lived in remote or very remote regions. Participants were surveyed for anxiety disorders (social phobia, GAD or panic disorder), mood disorders (dysthymia, minor depression and unipolar and bipolar major depression), AUDs (alcohol abuse and dependence) and psychological distress at baseline (2006–2009), 12 months, 3 years and 5 years	AUDIT CIDI V3.0 K10 PHQ-9	2006–2009	2,639	27%	Findings from the first ARMHS survey found that 29.2% of participants reported moderate levels of current distress on the K10 (score 16 +), which was deemed comparable to that reported for the 2007 NSMHWB [42]. ARMHS participants in outer regional and remote locations reported lower rates of psychological distress compared to inner regional respondents, while people in very remote areas reported elevated distress [44]. The rate of caseness (K10 scores > 15) was highest for participants in very remote areas [45, 46]
Canada	CCHS-MH (2012)	Members of the Canadian armed forces [48, 50]	The Canadian Community Health Survey-Mental Health and Well-being–Canadian Forces (CCHS-CF) was a cross-sectional survey of members of the regular forces and reservists who have paraded within the past six months. The survey was conducted as a supplement to the 2002 CCHS-MH survey for the general population and collected data on anxiety disorders (GAD, panic disorder and social phobia), MDD, PTSD and alcohol dependence	CIDI V2.1	2002	8,441	80.8%	Members of the regular forces had higher 12-month prevalence rates of MDD when compared to similar subsamples of the 2002 and 2012 general population surveys (7.97% vs. 3.50% and 3.48%, respectively) [48]. Age and sex-matched comparisons also found similarly higher rates of 12-month depression for the 2002 regular forces compared to the 2002 general population (7.6% vs. 4.3%), and increased rates of panic disorder (2.2% vs. 1.4%) [50]
		Members of the Canadian armed forces [47–49]	The 2013 Canadian Forces Mental Health Survey (CFMHS) was a cross-sectional survey of members of the regular forces and reservists who had been deployed to Afghanistan. The survey collected data on anxiety disorders (GAD, panic disorder and social phobia), MDD, PTSD and alcohol dependence	CIDI V3.0	2013	8,161	79.8%	Members of the regular forces had higher 12-month prevalence rates of MDD when compared to similar subsamples of the 2012 general population survey (7.96% vs. 3.48%) [48]. Lifetime and 12-month rates for GAD were also significantly higher among members of the regular forces compared to the general population (12.1% and 4.7% vs 9.5% and 3.0%, respectively) [49]
England	APMS (2014)	Ethnic minority populations [51]	The Study of Ethnic Minority Psychiatric Illness Rates in the Community (EMPIRIC) was a cross-sectional survey of people aged 16–74 who agreed to be re-contacted following the 1999 Health Survey for England and belonged to one of the following ethnic minority populations in England: Black Caribbean, Indian, Pakistani, Bangladeshi and Irish people. Interviews were conducted in the respondent’s first language and the results translated back into English where required. The survey collected data on common mental disorders (CMDs) including somatic symptoms, anxiety disorders (GAD, OCD, panic disorder and phobias), depressive episodes, mixed anxiety and depression, and psychosis	CIS-R PSQ	2000	4,281	68.2%	The rate of past week CMDs for White respondents was similar to those reported for the 1993 and 2000 Psychiatric Morbidity Surveys. Irish men, Pakistani women and Indian women had significantly higher overall rates of CMDs compared to White counterparts of the same gender, while the rates for Bangladeshi women were significantly lower. After adjusting for age, only the differences for Bangladeshi women were significant. Compared to White respondents, rates of 12-month psychosis were higher in the Black Caribbean and Pakistani groups, but lower in the Bangladeshi group. Somatic symptom scores were higher for Bangladeshi men and South Asian women. CMDs were more common among those interviewed in English, but the difference was only significant for the Bangladeshi group and was higher for women
		Homeless adults [52, 62]	The Survey of Psychiatric Morbidity among Homeless People was conducted as part of a suite of surveys that began with the 1993 Adult Psychiatric Morbidity Survey. The survey population included homeless people who were sleeping rough and visiting day centres, people staying in night shelters, residents of hostels for homeless people and people living in temporary housing accommodation in Great Britain. The survey collected data on anxiety disorders (GAD, OCD, panic disorders and phobias), depressive episodes, mixed anxiety and depression, alcohol dependence, drug dependence and psychotic disorders	CIS-R GHQ	1994	1,166	63.9%	The proportion of adults with common mental disorders (CMDs) (CIS-R score ≥ 12) was significantly higher for residents of hostels (38%) and people living in temporary housing (35%) compared to the general population (14%) [52]. Similarly, using the higher cut-off (CIS-R score ≥ 18) yielded prevalence rates of 28% and 27% in those respective homeless populations compared to 7% in the 1993 household survey [52]. The prevalence of psychotic disorders was also higher, estimated to be 8% and 2% compared to 1%, respectively [62]. Approximately 60% of people using day centres and those staying in night shelters had GHQ scores at or above the threshold of psychiatric caseness (GHQ score ≥ 4), and approximately 40% of those visiting day centres and 47% of those staying in night shelters met the criteria for the higher cut-off (GHQ score ≥ 6) [52]. Alcohol and non-cannabinoid drug dependence were also higher among night shelter residents (44% and 22%) and day centre visitors (50% and 13%) compared to the general population (5% and 3%) [52]
		Prisoners and young offenders [53]	The ONS Survey of Psychiatric Morbidity among Prisoners was a cross-sectional survey of female and male sentenced and remand prisoners aged 16–64 years in England and Wales. It was conducted as part of the suite of psychiatric surveys that began with the 1993 APMS. The survey collected data on neurotic, stress-related and somatoform disorders (phobias, panic disorder, GAD, mixed anxiety and depressive disorder, OCD and PTSD), depressive episode, manic episode, BP, schizophrenia and other non-organic functional psychoses, AUDs, drug dependence (cannabis, amphetamines, crack, cocaine, ecstasy, tranquillisers, opiates and volatile substances) and personality disorders	AUDIT CIS-R DIS (5 questions) SCAN V1.0 SCID-II	1997	3,142	88%	The survey found CIS-R scores indicative of significant neurotic symptoms (CIS-R score ≥ 12) for 39% of sentenced male prisoners, 58% of male remand prisoners, 62% of sentenced female prisoners and 75% of female remand prisoners compared to 12% of males and 18% of females in the general population survey. Prevalence rates for any 12-month functional psychosis were 7% for sentenced male prisoners, 10% for male remand prisoners and 14% for female prisoners compared to 0.4% in the general population survey
		Residents of institutions catering to people with mental illness [54]	The Survey of Psychiatric Morbidity among Adults Living in Institutions was conducted as part of a suite of surveys that began with the 1993 APMS. The survey population included people aged 16–64 permanently residing (≥ 6 months) in accommodation for people with mental illness in Great Britain. Relevant settings included hospitals, residential care homes, alternative residential accommodation, and institutions for the long-term care of people with mental disorders. The survey collected data on neurotic psychopathology and primary diagnosis including organic mental disorders, schizophrenia, delusional disorders, schizoaffective disorders, affective psychoses including mania and BP, and neurotic, stress-related and somatoform disorders. Proxy informants (e.g. doctors, nurses) were used where required	CIS-R	1994	1,191	N/A	The survey determined that 70% of all residents had schizophrenia, delusional or schizoaffective disorders as a primary diagnosis, 8% had affective psychoses, 8% had neurotic disorders, 2% had organic mental disorders and 2% had other mental disorders. Schizophrenia, delusional and schizoaffective disorders, affective psychoses and organic mental disorders were all more common in hospital settings, while neurotic disorders were more prevalent in residential settings The proportion of people in psychiatric hospitals with neurotic disorders was considered relatively low given that people with these disorders reported admissions in the 1993 household survey. It was hypothesised that the typical hospital stay might be short for most people with neurotic disorders, thus excluding them from the institutional survey
The United States	NSDUH (2014)	Active-duty military personnel (non-deployed) [55, 56]	The Department of Defence Health Related Behaviors Survey of Active-Duty Military Personnel was a cross-sectional survey of non-deployed active-duty soldiers of the United States military. This included members of the Army, Navy, Airforce and Marine Corp, collectively reported on as the Department of Defence (DoD), and separately, members of the Coast Guard. The study collected data on anxiety, depression, PTSD and psychological distress	BTQ K6 PHQ-9 PTSD checklist	2011	154,011 DoD; 5,461 Coast Guard	22% DoD; 37% Coast Guard	Of surveyed personnel, 10% reported high-level symptoms of depression in the past week and 5% experienced high posttraumatic stress levels based on symptoms they were experiencing
		Active-duty soldiers [57]	The Army STARRS All Army Study (AAS) is a cross-sectional survey of active-duty soldiers, exclusive of those in Basic Combat Training or deployed to a combat theatre. The survey ran from April to December 2011 and collected data on MDD, BP I and II or subthreshold BP, GAD, panic disorder, PTSD, ADHD, IED and SUDs	CIDI-SC PTSD checklist	2011	5,428	49.8%	Prevalence estimates from the AAS were higher than those in a calibrated civilian sample from the National Comorbidity Study - Replication (NCS-R), a previous household survey conducted using the CIDI in 2001 – 2003. Findings were not compared to the 2014 NSDUH
		Federal prison and jail inmates, and inmates in ‘special facilities’ [58, 59]	The National Inmate Survey (NIS-3) is a cross-sectional survey data of adults (18 + years) in Federal prison, jail, and ‘special facilities’ (e.g. prison hospitals, prison farms, boot camps). Data was collected between February 2011 and May 2012. The survey collected data on severe psychological distress (SPD)	K6	2011–2012	106,532	60% prison inmates; 61% jail inmates	As compared to the general population (2009 – 2012 NSDUH), the percentage of jail inmates who met the threshold for SPD (26%) was five times higher than the percentage of the general US population (5%) or those in the general US population with no criminal involvement in the past year (4%). The percentage of jail inmates who met the threshold for SPD was double the general population who were on probation or parole (11%) or who had been arrested in the past year (14%). Findings were not compared to the 2014 NSDUH
		State and federal correctional facility inmates [60, 61]	The Survey of Inmates in State and Federal Correctional Facilities is a cross-sectional survey of state and federal inmates in the USA. The survey collected data on symptoms of mental illness (i.e. major depression, mania, or psychotic disorders) and self-reported 12-month diagnoses	Modified structured clinical interview using the DSM-IV	2004	14,499 state; 3,686 federal	10.2% state; 13.3% federal	Of the inmates surveyed 49% of state inmates, and 40% of federal inmates, experienced symptoms of mental illness (i.e. major depression, mania, or psychosis) in the past 12-months. Additionally, 9% of state inmates, and 5% of federal inmates, reported being told by a health professional that they had a mental illness in the year before arrest or since admission. Prevalence findings could not be compared to the civilian population estimates from the NSDUH because of differences in measurement

*AAS* Army STARRS All Army Study, *ADHD*: attention-deficit hyperactivity disorder, *APMS* Adult Psychiatric Morbidity Survey, *ARMHS* Australian Rural Mental Health Study, *AUD* alcohol use disorder, *AUDIT* Alcohol Use Disorders Identification Test, *BP* bipolar disorder, *BTQ* Brief Trauma Questionnaire, *CCHS-CF* the Canadian Community Health Survey-Mental Health and Well-being – Canadian forces, *CCHS-MH*: Canadian Community Health Survey-Mental Health, *CFMHS*: Canadian Forces Mental Health Survey, *CIDI-SC* Composite International Diagnostic Interview Screening Scales, *CIDI V2.1* Composite International Diagnostic Interview, version 2.1, *CIDI V3.0* Composite International Diagnostic Interview, version 3.0, *CIS-R*: Clinical Interview Schedule -Revised, *CMD*: common mental disorder, DIS: Diagnostic Interview Schedule, *DoD*: Department of Defence, *DSM-IV*: Diagnostic and Statistical Manual of Mental Disorders, fourth edition, *EMPIRIC*: study of Ethnic Minority Psychiatric Illness Rates in the Community, *GAD*: generalised anxiety disorder, *GHQ*: General Health Questionnaire, *K6*: the Kessler Psychological Distress Scale-6, *K10*: Kessler Psychological Distress Scale-10, *IED* intermittent explosive disorder, *MDD* major depressive disorder, *NSDUH* National Survey of Drug Use and Health, *NSMHWB* National Survey of Mental Health and Wellbeing, *PHQ-9*: Patient Health Questionnaire-9, *PSQ* Psychosis Screening Questionnaire, *PTSD*: posttraumatic stress disorder, *OCD* obsessive compulsive disorder, *ONS* Office for National Statistics, *SCAN V1.0*: Schedule for Clinical Assessment in Neuropsychiatry, version 1.0, *SCID-II*: Structured Clinical Interview for DSM-IV Axis II Disorders, *SPD* severe psychological distress, *SUDs* substance use disorders, *US* United States

Supplementary survey findings from Australia showed that while the overall rate of moderate psychological distress in rural and remote areas was similar to that reported for the general household survey [42], distress scores indicating caseness were >10% more prevalent in very remote locations relative to other rural and remote regions [46]. Data from the supplementary surveys conducted in Canada showed that major depressive disorder, generalised anxiety disorder and panic disorder were more prevalent in Canadian military personnel than in members of the civilian population [47–50]. While there were no direct comparisons between the military surveys and the 2014 NSDUH findings in the USA, comparisons made to earlier survey results in the USA also indicated higher rates of mental disorders in active-duty soldiers [55, 56].

The supplementary survey of people in correctional facilities in England and Wales showed that significant neurotic symptoms and functional psychosis were far more common among prisoners than members of the general population [53]. While comparisons between surveys in the USA were limited by differences in the measures used, the results from the National Inmate Survey (NIS-3) also showed significantly higher rates of severe psychological distress among inmates when compared to the general population [58, 59].

The supplementary survey of homeless people conducted in Great Britain showed that the prevalence rates of common mental disorders, psychotic disorders, and alcohol and non-cannabinoid drug dependence were significantly higher in this group than the general population [52, 62]. In contrast, the supplementary survey of ethnic minority populations found that common mental disorders were more prevalent in people who were interviewed in English and would therefore not have been excluded from the general household survey [51].

Lastly, the supplementary survey of residents of institutions catering to people with mental illness in Great Britain showed a high proportion of people with severe disorders including schizophrenia, delusional disorder, schizoaffective disorder and affective psychoses, particularly in hospital settings [54]. Neurotic disorders, however, were more common in residential settings and comparisons to the general household survey data suggest that while people with these disorders do spend time in hospital, their length of stay in those facilities is likely to be relatively short in most cases [54].

### Supplementary surveys of non-responders

The 2007 National Survey of Mental Health and Wellbeing (NSMHWB) in Australia and the Netherlands Mental Health Survey and Incidence Study (NEMESIS-2) attempted to contact non-responders for information on their mental health (Table 4). In both instances, an abridged version of the original survey was used, which allowed for some comparison between responders and non-responders [11, 13, 19].

**Table 4:** Non-response surveys and response-based analyses

Country	Primary survey (year)	Supplementary survey target population	Description of supplementary survey or analysis	Instrument(s)	Year	Sample	Response rate	Findings
Australia	NSMHWB (2007)	Non-responders to the 2007 NSMHWB [13]	A Non-Response Follow-Up Study (NRFUS) was conducted in Sydney and Perth based on reduced survey content. The survey collected demographic information and data on symptoms of psychological distress	K10	2008	151	39%	Findings from the NRFUS yielded higher prevalence rates of psychological distress for people surveyed in Perth, males and younger people when compared to the results of the NSMHWB, while the results from Sydney showed lower prevalence. The unweighted K10 score for the NRFUS was 15.6. Applying the NRFUS K10 scores more broadly to the non-respondents of the 2007 NSMHWB yielded a revised score of 14.8, which was not deemed to be significantly different from the original score of 14.4. Given the small size of the NRFUS, the results were not incorporated into the 2007 NSMHWB strategy
Japan	WMHJ2 (2013–15)	Regions with varying response rates [18]	Researchers conducted an analysis of the association between area response rate and prevalence estimates of common mental disorders in the 129 areas sampled for the WMHJ2. Prevalence estimates were also compared between two surveys with different response rates conducted in the same area in different years	CIDI V3.0	2013–2015	2,450	5–80%	Response rate ranged from 5 to 80 between regions and was not associated with mental disorder prevalence across the 129 areas surveyed. Prevalence of mental and SUDs were also significantly lower in the same area when survey response was higher
The Netherlands	NEMESIS-2 (2007–9)	Non-responders to the NEMESIS-2 survey [19]	All eligible non-responders to the NEMESIS-2 were re-contacted to participate in a shorter survey. The survey collected data on symptoms of depression and anxiety, and symptoms of childhood impulse control disorders	MHI CIDI V3.0 (4 items)	2007–2009	1,229	26.1%	After controlling for demographic variables, non-responders were 1.75 times more likely than responders to have had mood and anxiety problems in the past four weeks, and 2.04 times more likely to have had at least one impulse control symptom during childhood. Both differences were statistically significant

*CIDI V3.0*: Composite International Diagnostic Interview, version 3.0, *K10* Kessler Psychological Distress scale-10, *MHI*: Mental Health Inventory, *NEMESIS-2* The Netherlands Mental Health Survey and Incidence Study-2, *NRFUS*: Non-response Follow-Up Study, *NSMHWB* National Survey of Mental Health and Wellbeing, *SUDs* substance use disorders, *WMHJ2* World Mental Health Japan 2^nd^ Survey

The NSMHWB Non-response Follow-up Survey (NRFUS) found that psychological distress, as measured by the Kessler Psychological Distress Scale (K10), was higher among non-responders than in the responding NSMHWB sample [13]. However, applying the NRFUS scores to the broader population of people who did not respond to the NSMHWB did not significantly increase the overall K10 score for the survey [13]. The NEMESIS-2 non-response survey found that compared to responders, non-responders were significantly more likely to have recent mood and anxiety problems as well as at least one impulse control symptom in childhood [19]. Despite the reduced survey content, participation rates for these follow-up surveys remained poor, with only 26.1% [19] to 40% [11, 13] of people who were contacted responding.

Another survey in Japan, the WMHJ2, analysed the association between response rate and mental disorder prevalence in different regions and conducted additional sampling of an area with a low response rate [18]. The findings showed no association between disorder prevalence and regional response rate; a lower disorder prevalence was reported for the area that was re-sampled when a higher response rate was achieved [18].

## Discussion

Our review of national mental health surveys found that people not living in private dwellings in the community, especially those who are homeless, in health or correctional facilities, were commonly excluded from sampling, while males and young people were commonly underrepresented among survey respondents. Supplementary surveys of excluded populations and non-responders, where available, indicated higher prevalence of mental health problems in many of these groups. The common exclusion of key vulnerable groups and relatively high rates of non-response in these surveys have important implications for how results are used and interpreted.

The findings of this review show that most national mental health surveys recently conducted in high-income countries employ a similar approach and therefore have similar limitations concerning excluded populations and non-responders. This is not entirely surprising as six of the surveys included for review were either part of the WMH Survey Initiative or rooted in a previous WMH survey conducted in that country [11, 13, 18, 22–26]. Furthermore, given the recognition and prominence of the WMH Initiative, other independent surveys may have adopted similar sampling methodologies.

Four surveys included for review explicitly attempted to quantify the proportion of the population that would be excluded according to their methodology, with estimates ranging between 1% and 3.5% [15, 20, 25, 27, 29]. However, since countries may differ considerably with respect to the size of some excluded groups (e.g. military personnel, non-local language speakers), these figures may not be more widely representative. While the exclusion of such a small proportion of the total population may have had little to no impact on the overall survey results, as was sometimes articulated in the literature [19], excluded groups may have accounted for a far larger proportion of people in a particular subpopulation with specific risk factors and service needs (e.g. minority group, age cohort, or clinical severity group) [11, 63, 64]. In that instance, a difference in disease prevalence between included and excluded individuals could significantly affect the accuracy of disorder and service need estimates generated for that subpopulation. It may also impact the estimates of disease burden, particularly where individuals with more severe disorders are undersampled.

For example, data from 12 high-income OECD countries in 2019 showed that the proportion of people 80 years of age or older receiving care in long-term (i.e. residential) facilities (excluding hospitals) ranged between 10-19% [65]. Therefore, while this group may represent a relatively small proportion of a country’s total population, they would account for a far larger proportion of the elderly population. Research also suggests that mental disorders are more common among people residing in residential care relative to their community-dwelling counterparts [66, 67], yet only one of the surveys that met our inclusion criteria sampled this population [27, 29]. The exclusion of people in residential care from surveys may therefore result in an underestimate of both the disorder prevalence for this age group and the resources required to meet the needs of those living in these settings. As the populations in high-income countries continue to age, the impact of this group’s exclusion is likely to become even more profound [68].

Data from the supplementary surveys indicated that many of the other groups commonly excluded from population-based mental health surveys are also especially vulnerable to psychological distress and mental illness, including prisoner populations [53, 58, 59], military personnel [48–50, 57], homeless people [52, 62], people in hospitals and other health facilities [54], and people living in very remote areas [44–46]. It is particularly important to note the small number of surveys that included psychotic and personality disorders as well as the overrepresentation of individuals with a severe disorder among homeless populations [52, 62], prisoner populations [53] and people in hospitals and health facilities [54]. Individuals with severe mental disorders have high service needs, with estimates from England indicating that hospital admissions for these disorders account for 7% of total bed days for all health conditions [69, 70]. It is therefore imperative that service planning adequately account for the needs of these groups. One approach to gaining a better understanding of the size, distribution and needs of these populations is by conducting surveys through existing mental health services, like the Survey of High Impact Psychosis in Australia [71] and its predecessor the Study on Low Prevalence Disorders [72]. In countries with patient registers like Denmark, data on these populations are instead typically derived from admissions data [73, 74], however both methodologies are limited in that they rely on individuals with severe disorders being in contact with health services.

The lack of clear criteria around the inclusion or exclusion of people with cognitive impairment was another important finding of this review. Research has shown that individuals with a cognitive impairment or intellectual disability have a higher prevalence of mental disorders than those without, and have more complex service needs as a result of their comorbid conditions [75, 76]. As such, ambiguity regarding the inclusion of these individuals in national mental health surveys may complicate attempts to interpret and use survey data with respect to understanding and planning for mental health services overall and specifically for this population.

Survey results may be skewed if non-responders have a different mental health profile. Recent research shows that the response rates for health surveys in high-income countries have generally declined over time [77–79] and this trend was noted where previous iterations of the mental health surveys included in this review could be identified [14, 15, 19, 80–84]. Few studies attempted to capture non-responders or people in excluded groups through supplementary surveys or additional survey rounds, thereby limiting the generalisability of their results. In some cases, supplementary surveys also used different mental health measures, thus preventing the direct comparison or integration of results to those of the broader population surveys.

Similar to previous findings, supplementary analyses of non-response did not show a consistent relationship between survey response and mental health status. The first NEMESIS survey conducted in 1996 found that non-responders who agreed to complete the 12-item General Health Questionnaire (GHQ-12) had slightly better average mental health scores when compared to those who participated in the full diagnostic interview [80]. On the other hand, high levels of mental distress were associated with increased rates of attrition from the Nord-Trøndelag Health Study (HUNT) in Norway [85] and previous psychiatric diagnoses were tied to lower rates of participation in the population-based PART (Psykisk hälsa, Arbete och RelaTioner) study on mental health in Sweden [86]. Finally, while considerable evidence has linked heavy alcohol consumption, alcohol-related problems and financial support or hospital treatment for substance use disorders to lower rates of survey response [85–90], the opposite relationship has also been found [91–94].

Strategies adopted for the purposes of improving response rates included financial incentives, i.e. money or vouchers (Response Rate (RR): 57.3-65.1%) [12, 19, 20, 25], pre-survey notification (RR: 43.4-80%) [12, 15, 18, 19, 20, 24, 25, 27, 28], follow-up contact attempts (RR: 57.3-80%) [13, 15, 19–21, 25–27] and interviewing or contacting people face-to-face (60- 65.1%) [13, 19]. Interestingly, the survey that was mailed to potential participants had one of the lowest response rates (44.3%) [21] of the surveys reviewed, while the survey with the highest response rate (80%) had one of the shortest mental health questionnaires [27, 29], supporting previous findings that shorter questionnaires improve participation [95].

The underrepresentation of males and young people in our findings has also been reported in other health and health-related surveys [96–100]. One of the surveys included in our review oversampled younger people to ensure adequate representation [11, 13]. No surveys specifically adjusted their sampling methodology to account for sex or gender; however, nearly all surveys applied sampling weights to adjust for lower response rates and reflect the demographics of the general population [11–29]. It is possible that there may also be other populations that are underrepresented in survey findings but that were not specifically mentioned in the related literature, such as shift workers, fly in–fly out workers and long-haul transport drivers, all of whom have been found to be at elevated risk of poor mental health [101–104]. Other more specific issues with survey sampling methodologies, such as those described by Kalton et al.[105], were outside the scope of this review.

Planners and policymakers should be aware of the limitations of the mental health surveys that they use to understand mental health service needs and resource requirements. Ideally, efforts to sample non-responders and previously excluded populations would be included in all national mental health survey designs, or in complementary studies that use a standardised methodology to allow direct comparisons. However, given the significant additional resources required, sampling these populations may not always be feasible. In such instances, end users of survey results could consider other available data on the level of need within excluded populations to adjust estimates where appropriate. Adjustments of this kind were recently made to the prevalence estimates informing the mental health service demand and resource requirement modelling for adequate care in Australia’s NMHSPF (more information available in the NMHSPF V4.1 Technical Appendices [4]). Behan and Kennelly [106] used a similar approach to incorporate homeless and prison populations into their estimates for the cost of schizophrenia in Ireland. Given the increasing rates of non-response, groups conducting future surveys may also want to give greater consideration to factors that could influence participation, such as survey length and method of delivery, in their survey design [95].

### Strengths and limitations 

The lack of information available on which groups were included and excluded from many of the surveys was a key finding and limitation of this review. It suggests that more consistent reporting of these criteria in survey methodologies would be highly beneficial to improving our understanding of prevalence rates and facilitating comparisons between surveys. Another important limitation of this review was that the search for supplementary surveys of non-responders and excluded populations was restricted to those explicitly referred to in the primary survey literature. As a result, other potentially relevant surveys would not have been identified. For example, the First Nations Regional Health Survey conducted in Canada between 2008 and 2010 sampled First Nations communities that would have been excluded from the national mental health survey, but was not identified through our search criteria [107]. Unfortunately, a broader search of supplementary surveys was not feasible given the large number of excluded groups that were identified. Finally, as the review was limited to surveys conducted in high-income OECD countries, the findings may not be applicable to other settings.

## Conclusion

This study showed that there are key populations that are often excluded from national mental health surveys in high-income countries (e.g. persons who are homeless, in hospitals or health facilities, or correctional institutions). The exclusion of these populations, and the few attempts to follow-up survey non-responders, may limit the generalisability of survey findings and result in underestimates of need for care. Collectively, our findings suggest the need for more inclusive sampling methods, or targeted population surveys, to strengthen the accuracy of prevalence estimates drawn from these surveys to inform policy and service planning decisions. If there are resource restrictions that limit the feasibility of these options, consideration should be given to whether prevalence estimates may be adjusted to account for any exclusions. Additionally, clear reporting on who is included and/or excluded in survey sampling procedures may ensure greater accuracy in the interpretation of survey findings.

## Data Availability

Data sharing is not applicable to this article as no new data were created or analysed in this study.
